# The Effects of COVID-19 Lockdown on Glycaemic Control and Lipid Profile in Patients with Type 2 Diabetes: A Systematic Review and Meta-Analysis

**DOI:** 10.3390/ijerph19031095

**Published:** 2022-01-19

**Authors:** Omorogieva Ojo, Xiao-Hua Wang, Osarhumwese Osaretin Ojo, Edith Orjih, Nivedita Pavithran, Amanda Rodrigues Amorim Adegboye, Qian-Qian Feng, Paul McCrone

**Affiliations:** 1 School of Health Sciences, Faculty of Education, Health and Human Sciences, Avery Hill Campus, University of Greenwich, Avery Hill Road, London SE9 2UG, UK; P.McCrone@greenwich.ac.uk; 2 The School of Nursing, Soochow University, Suzhou 215006, China; wangxiaohua@suda.edu.cn (X.-H.W.); 20195231027@stu.suda.edu.cn (Q.-Q.F.); 3 Smoking Cessation Department, University Hospital, South London and Maudsley NHS Foundation Trust, Lewisham High Street, London SE13 6LH, UK; osarhumwese.ojo@slam.nhs.uk; 4 Diabetes and Endocrine Department, Darent Valley Hospital, Dartford & Gravesham NHS Trust, Dartford DA2 8DA, UK; edith.orjih@nhs.net; 5 Department of Clinical Nutrition, Amrita Institute of Medical Sciences and Research Centre, Amrita Vishwa Vidyapeetham, Kochi 682041, India; ammasnivi@gmail.com; 6 Centre for Healthcare Research, School of Nursing, Midwifery and Health, Faculty of Health and Life Sciences, Coventry University, Priory Street, Coventry CV1 5FB, UK; ad6287@coventry.ac.uk

**Keywords:** type 2 diabetes, COVID-19, COVID-19 lockdown, coronavirus disease-2019, SARS-CoV-2, glycated haemoglobin, lipid parameters, body mass index

## Abstract

The impact of the COVID-19 lockdown on glycaemic control and other metabolic parameters in patients with type 2 diabetes is still evolving. Aim: This systematic review and meta-analysis aims to examine the effects of COVID-19 lockdown on glycaemic control and lipid profile in patients with type 2 diabetes. Methods: The PRISMA framework was the method used to conduct the systematic review and meta-analysis, and the search strategy was based on the population, intervention, control and outcome (PICO) model. The Health Sciences Research databases was accessed via EBSCO-host, and EMBASE were searched for relevant articles. Searches were conducted from inception of the databases until 17 September 2021. Results: The results identified three distinct areas: glycaemic control, lipid parameters and body mass index. It was found that COVID-19 lockdown led to a significant (*p* < 0.01) increase in the levels of glycated haemoglobin (%) compared with pre-COVID group (gp) with a mean difference of 0.34 (95% CI: 0.30, 0.38). Eleven studies contributed to the data for glycated haemoglobin analysis with a total of 16,895 participants (post-COVID-19 lockdown gp, *n* = 8417; pre-COVID gp, *n* = 8478). The meta-analysis of fasting plasma glucose (mg/dL) also showed a significant (*p* < 0.05) increase in levels of post-COVID-19 lockdown gp compared with pre-COVID gp, with a mean difference of 7.19 (95% CI: 5.28, 9.10). Six studies contributed to fasting plasma glucose analysis involving a total of 2327 participants (post-COVID-19 lockdown, *n* = 1159; pre-COVID gp, *n* = 1168). The body mass index (BMI) (kg/m^2^) analysis also demonstrated that post-COVID-19 lockdown gp had a significantly (*p* < 0.05) higher BMI than the pre-COVID gp with a mean difference of 1.13 (95% CI: 0.99; 1.28), involving six studies and a total of 2363 participants (post-COVID-19 lockdown gp, *n* = 1186; pre-COVID gp, *n* = 1177). There were significantly (*p* < 0.05) lower levels of total cholesterol (mmol/L), triglyceride (mmol/L) and LDL cholesterol (mmol/L), and higher levels of HDL cholesterol (mg/dL) in the post-COVID-19 lockdown gp compared with pre-COVID gp, although these results were not consistent following sensitivity analysis. Conclusion: The findings of the systematic review and meta-analysis have demonstrated that COVID-19 lockdown resulted in a significant increase (*p* < 0.05) in the levels of glycated haemoglobin, fasting glucose and body mass index in patients with type 2 diabetes. In contrast, the effect of the lockdown on lipid parameters, including total cholesterol, triglycerides, LDL and HDL cholesterol was not consistent.

## 1. Introduction

The coronavirus disease-2019 (COVID-19) has become a global pandemic and an international public health emergency [1]. The pandemic has challenged the healthcare system worldwide [2]. The disease may range from a mild acute respiratory illness to more severe pneumonia with associated respiratory failure, acute respiratory distress syndrome and septic shock [3]. It has been reported that older adults and those with underlying comorbidities, such as cardiovascular diseases and diabetes are at greater risk of a severe form of COVID-19 [3]. In fact, the COVID-19 pandemic is also regarded as a syndemic, where health determinants, including social determinants and comorbidities interact and cumulatively and adversely exacerbate the pre-existing disease burden and its unfavourable effects [2,4]. Furthermore, the effect of COVID-19 and its lockdown on access to diabetes care and glycaemic control in people with type 2 diabetes is still evolving [5]. There have been reports of dysregulation of glycaemic control leading to acute diabetic complications in patients with type 2 diabetes [6].

Therefore, exploring the effects of COVID-19 lockdown in patients with diabetes is especially significant against the backdrop of the high prevalence of diabetes, which is on the increase globally [7]. Diabetes is a major cause of morbidity and mortality and leads to a significant health and financial burden worldwide [8]. The global prevalence of diabetes is estimated to rise to 700 million by 2045, from 463 million in 2019 who were living with the disease [7]. On the other hand, the COVID-19 pandemic has led to more than 100 million infections and more than two million deaths globally, as of 20 February 2021 [9].

### 1.1. Description of COVID-19 and Its Lockdown

COVID-19 is caused by the novel coronavirus, which has been named as severe acute respiratory syndrome (SARS-CoV-2) [10]. It was first identified in Wuhan, China in 2019 and reached pandemic proportions in February, 2020 with all countries of the world now affected by the disease [10].

During the COVID-19 pandemic, various measures were put in place by different governments around the world to stem the spread of the infection. Lockdown measures varied from cities, regions and countries and included voluntary home curfews, travel restrictions and prohibition of public and social events [11]. Other lockdown strategies involved declaration of states of emergencies by governments and reduced outings by individuals and person to person contacts, while persons were requested to avoid settings with poor ventilation [11]. There were also cancellations, postponement and scaling down of large-scale events, and closure of primary, secondary and tertiary institutions [12].

During the lockdown, only essential activities were allowed and mobility for most people, including patients with type 2 diabetes, was restricted to purchase of food and medications, although online activities were encouraged [13]. Furthermore, most patients’ clinics were closed except for remote and emergency visits, while hospitals were dealing with large number of COVID-19 patients [13].

### 1.2. How COVID-19 and Its Lockdown May Affect Patients with Diabetes

It is well established that the management of blood glucose and other metabolic parameters are quite challenging for patients with diabetes and this can be exacerbated by changes in lifestyle and daily routines, such as diet, exercise, insulin adjustments, emotions, stress, social relations and working activities [13]. Changes in lifestyle, such as lack of physical activities and increased intake of diet may promote weight gain, which has implications for insulin sensitivity and glycaemic control [12].

Profound changes in daily life due to the COVID-19 lockdown can have a significant effect on physical and mental health [11]. In this regard, changes in behaviour patterns and daily life, including exercise levels, stress and anxiety influence self-management of diabetes and glycaemic control [11]. These changes have significant implications for clinical outcomes during the lockdown [9].

The outbreak of the COVID-19 pandemic has had a direct effect on patients with diabetes in terms of increased prevalence of acute diabetic complications and greater emergency in healthcare practice [14,15]. On the other hand, the pandemic has had an indirect effect with respect to the impact of the lockdown and social distancing measures on economics, social life and glycaemic control [14].

### 1.3. Why It Is Important to Do the Review

While a number of primary research studies, systematic reviews and meta-analyses have focused on the effect of diabetes on outcomes, including mortality in COVID-19 patients, limited attention has been paid to the metabolic disturbances caused by COVID-19 lockdown in patients with diabetes [16]. Although it has been hypothesised that the lockdown measures did not affect glycaemic control in patients with diabetes, there are contrasting studies globally suggesting that these measures either promoted or played a detrimental role in regulating glycaemia and other metabolic parameters in patients with type 2 diabetes [14]. A meta-analysis of observational studies conducted by Silverii et al. [17] focused only on glycaemic control in patients with type 1 and type 2 diabetes during the lockdown. In contrast, the current review focuses on patients with only type 2 diabetes and the effect of the lockdown on glycemic control and lipid profile. This is in recognition of the fact that there appears to be limited data on the impact of the lockdown on glycaemic control and other metabolic parameters in patients with type 2 diabetes [13].

### 1.4. Aim

This systematic review and meta-analysis aims to examine the effects of COVID-19 lockdown on glycaemic control and lipid profile in patients with type 2 diabetes.

## 2. Methods

This systematic review and meta-analysis was conducted in accordance with the preferred reporting items for systematic review and meta-analysis (PRISMA) [18].

### 2.1. Types of Studies

The studies included in this review were cross-sectional, retrospective, prospective and case control studies.

### 2.2. Types of Participants

Participants selected for the review were those with type 2 diabetes irrespective of co-morbidities.

### 2.3. Types of Exposure

The studies included were those comparing parameters of interest in patients with type 2 diabetes during the pre-COVID-19 lockdown and post-COVID-19 lockdown. There were no restrictions in terms of the length of the COVID-19 lockdown.

### 2.4. The Inclusion Criteria

Studies involving participants with type 2 diabetes during pre- and post-COVID-19 lockdown were included in this review. In particular, studies that included such outcomes of interest as glycaemic control, lipid profile and anthropometric measurements were also included in this review.

### 2.5. The Exclusion Criteria

Studies involving patients with type 1 diabetes, prediabetes, gestational diabetes, healthy population and without outcomes of interest were excluded from the review. In addition, letters to editors were also excluded.

#### 2.5.1. Types of Outcome Measures

The following were the primary outcome measures of interest:

- Blood Glucose Parameters: Glycated haemoglobin (HbA1c, %);
- Lipid parameters: high-density lipoprotein (HDL mg/dL) cholesterol, total cholesterol (mmol/L), low-density lipoprotein (LDL mmol/L) cholesterol, triglycerides (mmol/L);
- Body mass index (BMI) (kg/m^2^).

#### 2.5.2. Secondary Outcome Measures of Interest

- Fasting Blood Glucose (FBG mg/dL);
- Postprandial Blood Glucose (mg/dL).

### 2.6. Search Methods for Identification of Studies

EMBASE and the Health Sciences Research databases (including MEDLINE, Academic Search Premier, APA PsycInfo, Psychology and Behavioural Sciences Collection, APA PsycArticles databases, and CINAHL Plus with Full Text) accessed via EBSCO-host were searched for relevant articles. The search method was based on the population, intervention, control and outcome (PICO) model (Table 1). The searches were conducted from inception of the databases until 17 September 2021. Medical subject headings (MesH) and synonyms were used as search terms and these were combined using Boolean operators (OR/AND). Two members of the group (OO and OOO) independently caried out the searches and these were cross-checked by the other members of the team. The results of the searches were exported to EndNote (Analytics, Philadelphia, PA, USA), where the duplicates were removed.

## 3. Data Collection and Analysis

### 3.1. Selection of Studies

**Figure 1:**
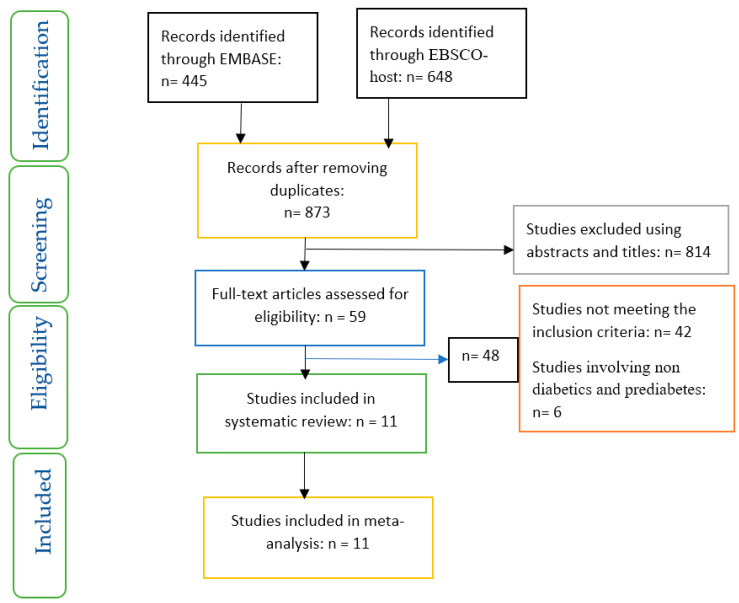
PRISMA flow chart on selection and inclusion of studies.

The studies included were based on a set of inclusion and exclusion criteria and these are represented in a PRISMA flow chart (Figure 1).

### 3.2. Data Extraction and Management

The following information was extracted from the studies included: the country of study, population characteristics (such as mean age), sample size, study design/methods and results (Table 2).

The data were extracted by two researchers (EO and NP) from the articles included and the information was cross-checked by another member (OO) of the research team. Final values were used to compare pre- and post-COVID-19 patients with type 2 diabetes.

### 3.3. Data Analysis

A meta-analysis was performed whenever there were enough studies reporting data on the same outcome of interest. Mean difference (MD) with 95% CIs. was used for analysis of continuous data and forest plots were used to illustrate the results of the meta-analysis. The statistical significance of the overall effect of the exposure was at *p* < 0.05.

Sensitivity analysis was carried out by removing studies one by one from the meta-analysis in order to examine the level of consistency of the results. On the other hand, the degree of heterogeneity of the included studies was measured using the *I^2^* statistic which is expressed as percentage [19]. For the outcomes measured, a fixed-effects model was used for the meta-analysis and whenever there was substantial heterogeneity (≥50%) and when there were enough studies included for the outcome, subgroup analysis (involving retrospective prospective, cross-sectional and case control studies) was conducted. In addition, final values were used to compare the pre-COVID-19 with the post-COVID-19 groups [19]. The meta-analysis was conducted using Review Manager (RevMan) 5.3 software [20]. In some of the outcomes of interest, the units of measurements were converted to ensure the same unit of measurements for all the studies included for that parameter. In studies reporting values in median, and 1st and 3rd quartiles, these were converted to means and standard deviations. In three of the included studies [14,21,22], the authors were not clear about the method of presentation of the relevant data (that is, whether they used mean ± SD or SEM or used Median (Minimum–Maximum) or Median (Interquartile range)). We sent emails to the corresponding authors for clarification and the following were confirmed by the corresponding authors: Biamonte et al. [22], data are presented as mean ± SD; D’Onofrio et al. [14], data are presented as median (25–75 percentiles); Ghosh et al. [21], data are presented as mean ± SD. 

## 4. Results

Eleven studies were included in the systematic review and meta-analysis. Three studies each were conducted in India [21,28,29] and Italy [14,22,23], while one each was conducted in France [24], Japan [25], Morocco [9], South Korea [27] and Turkey [26]. Six of these studies were retrospective, two each were prospective and cross sectional, while only one study was case control.

### 4.1. Assessment of Risk of Bias in Included Studies

**Figure 2:**
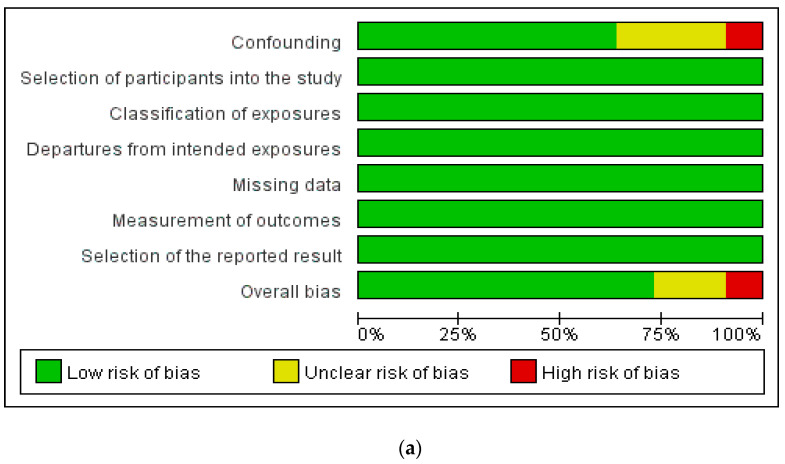

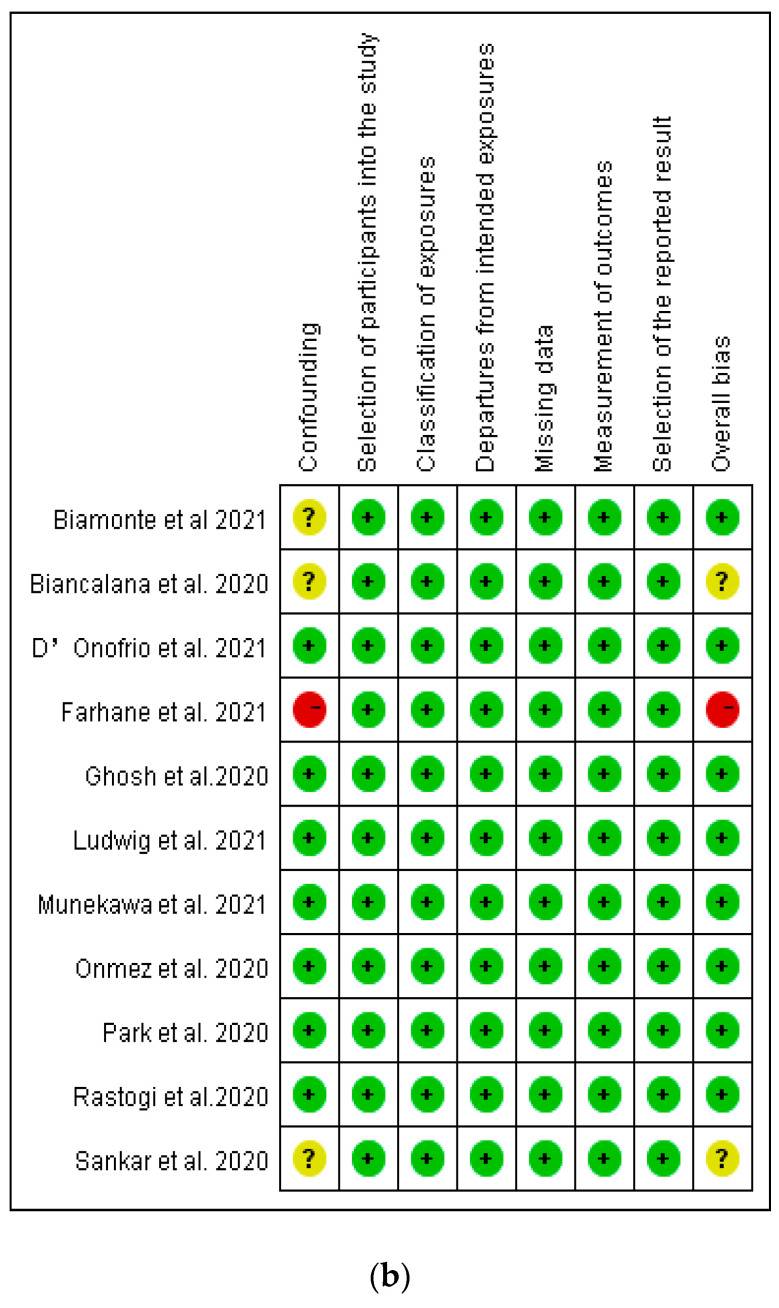
Shows (**a**) risk of bias graph and (**b**) risk of bias summary.

Two researchers (QF, XW) evaluated the risk of bias of included studies using the Preliminary Tool for Risk of Bias in Exposure Studies [30]. The domains assessed were overall bias, selection of the reported result, measurement of the outcome, missing outcome data, departures from intended exposures, classification of exposures, selection of participants into the study and bias due to confounding (Figure 2a,b). Seven studies [14,21,24,25,26,27,28] showed low risk of bias in all the domains assessed, while three studies [22,23,29] were of concern in bias due to confounding. One study [9] was assessed as having high risk with respect to bias due to confounding and was graded as having high overall risk.

The results of the systematic review and meta-analysis identified three distinct areas: glycaemic control, lipid parameters and body mass index.

### 4.2. Glycaemic Control

The results of the systematic review are outlined in Table 2. In particular, it was shown that COVID-19 lockdown either significantly (*p* < 0.05) increased glycated haemoglobin (HbA1c) (%) in patients with type 2 diabetes in some studies [9,22,25], did not change HbA1c significantly [14,26,29] or that there was overall improvement in glycaemic control [24,28].

**Figure 3:**
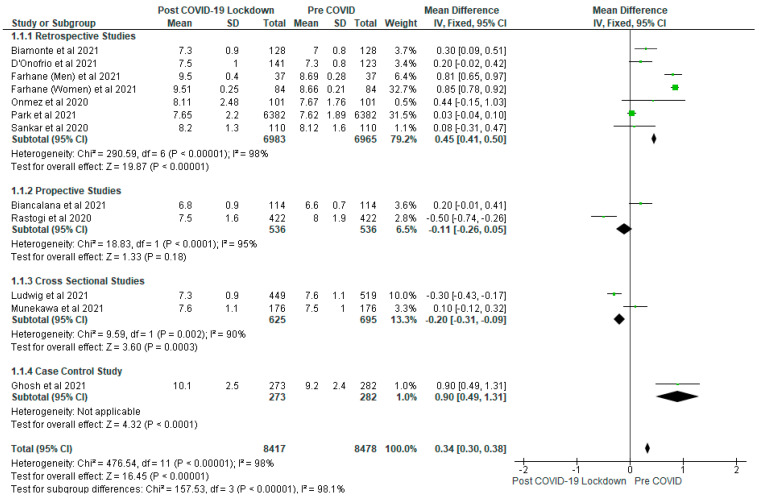
The effect of COVID-19 lockdown on Glycated Haemoglobin (%).

**Figure 4:**
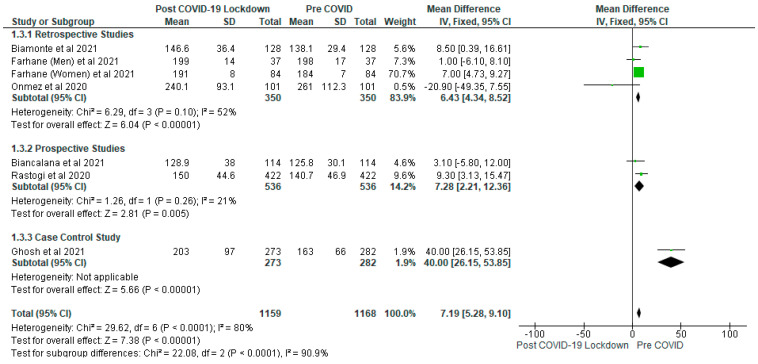
The effect of COVID-19 lockdown on Fasting Plasma Glucose (mg/dL).

**Figure 5:**
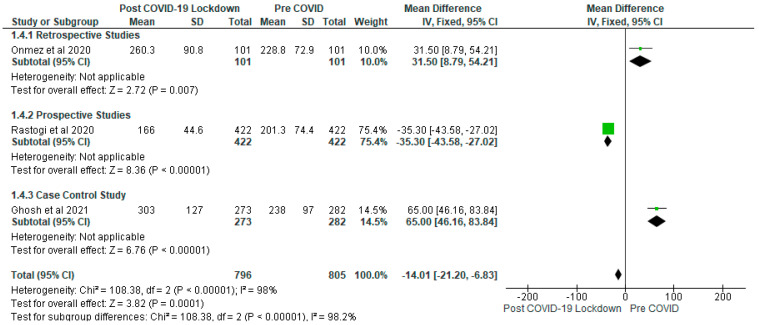
The effect of COVID-19 lockdown on Postprandial Blood Glucose (mg/dL).

The results of the meta-analysis on glycaemic control are shown in Figure 3, Figure 4 and Figure 5. It was found that COVID-19 lockdown led to a significant (*p* < 0.01) increase in the levels of glycated haemoglobin compared with the pre-COVID group (gp) with a mean difference of 0.34 (95% CI: 0.30, 0.38) (Figure 3). Following sensitivity analysis, this result remained consistent except when the Farhan et al. [9] study was removed from the meta-analysis that the result showed no significant differences (*p* > 0.05). Eleven studies contributed to the data for glycated haemoglobin analysis with a total of 16,895 participants (post-COVID-19 lockdown gp, *n* = 8417; pre-COVID gp, *n* = 8478). The results of the subgroup analysis showed that glycated haemoglobin was significantly (*p* < 0.05) increased in post-COVID-19 lockdown gp in the retrospective studies and case control study (Figure 3).

Biamonte et al. [22] showed that there was significant increase in the fasting plasma glucose in patients with type 2 diabetes following the COVID-19 lockdown. The meta-analysis of fasting plasma glucose also showed significant (*p* < 0.05) increase in levels of post-COVID-19 lockdown gp compared with pre-COVID gp with a mean difference of 7.19 (95% CI: 5.28, 9.10) (Figure 4). The finding remained consistent when each study was removed one by one from the meta-analysis. Six studies contributed to fasting plasma glucose analysis involving a total of 2327 participants (post-COVID-19 lockdown, *n* = 1159; pre-COVID gp, *n* = 1168). In terms of the subgroup analysis, the levels of fasting plasma glucose (mg/dL) were significantly (*p* < 0.05) increased in the post-COVID-19 lockdown gp compared to pre-COVID gp in all the subgroups.

In contrast, the results of the meta-analysis of postprandial blood glucose showed that post-COVID-19 lockdown gp had significantly (*p* < 0.05) reduced postprandial blood glucose (mg/dL) compared with the pre-COVID gp (Figure 5) with a mean difference of −14.01 (95% CI: −21. 20, −6.83) (Figure 5). Following sensitivity analysis, the result remained consistent except when Rastogi et al. [28] study was removed from the meta-analysis that the post-COVID-19 gp showed significantly (*p* < 0.05) higher postprandial blood glucose than the pre-COVID gp. Only three studies contributed to the analysis for postprandial blood glucose with 1601 participants involved (post-COVID-19 lockdown gp, *n* = 796; pre-COVID gp, *n* = 805). The subgroup analysis showed significantly (*p* < 0.05) higher levels of postprandial blood glucose in the post-COVID-19 lockdown gp in both the retrospective and case control studies. However, significantly (*p* < 0.05) lower levels were observed in the prospective study (Figure 5).

### 4.3. Lipid Parameters

**Figure 6:**
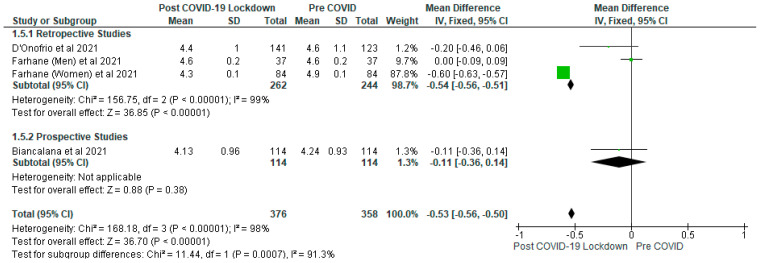
The effect of COVID-19 lockdown on Total Cholesterol (mmol/L).

**Figure 7:**
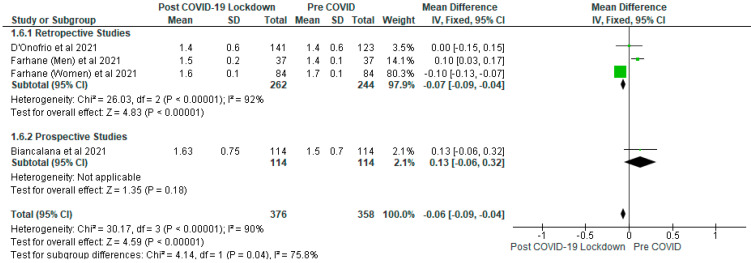
The effect of COVID-19 lockdown on Triglyceride (mmol/L).

**Figure 8:**
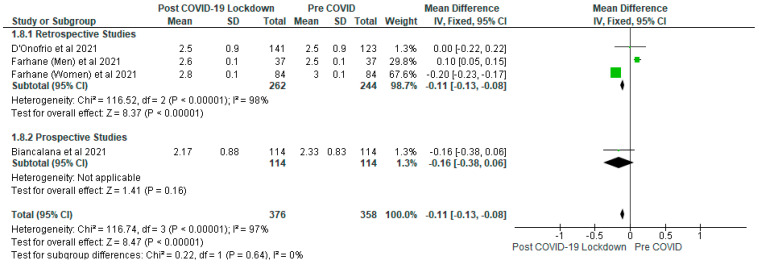
The effect of COVID-19 lockdown on LDL Cholesterol (mmol/L).

The meta-analysis showed that there were significantly (*p* < 0.05) lower levels of total cholesterol (mmol/L) (Figure 6), triglyceride (mmol/L) (Figure 7) and LDL cholesterol (mmol/L) (Figure 8) in the post-COVID-19 lockdown gp compared with pre-COVID gp. In addition, the sensitivity analysis revealed that when the Farhane et al. [9] study was removed from the meta-analysis, there was no significant difference (*p* > 0.05) between the post-COVID-19 gp and pre-COVID gp with respect to total cholesterol (mmol/L), triglyceride (mmol/L), LDL (mmol/L) and HDL cholesterol (mg/dL).

For total cholesterol (mmol/L), the mean difference was −0.53 (95% CI: −0.56, −0.50). Three studies were included in this analysis, with 734 participants (post-COVID-19 lockdown gp, *n* = 376; pre-COVID gp, *n* = 358). The subgroup meta-analysis showed that although there were significantly (*p* < 0.05) lower total cholesterol in the post-COVID-19 lockdown gp compared with pre-COVID gp in relation to the retrospective studies, the difference was not significant (*p* > 0.05) in the prospective study.

With respect to triglyceride (mmol/L), the mean difference between the post-COVID-19 lockdown gp and pre-COVID gp was −0.06 (95% CI: −0.09, −0.04), involving 3 studies and 734 participants (post-COVID-19 lockdown gp, *n* = 376; pre-COVID, *n* = 358) (Figure 7).

For LDL cholesterol (mmol/L), the mean difference was −0.11 (95% CI: −0.13, −0.08) and included four studies with 734 participants (post-COVID-19 lockdown gp, *n* = 376; pre-COVID, *n* = 358) (Figure 8). The subgroup analysis for triglyceride and LDL cholesterol showed significantly (*p* < 0.05) lower levels of these metabolites in the post-COVID-19 lockdown gp compared with pre-COVID gp in the retrospective studies while the differences were not significant (*p* > 0.05) in the prospective studies (Figure 7 and Figure 8).

**Figure 9:**
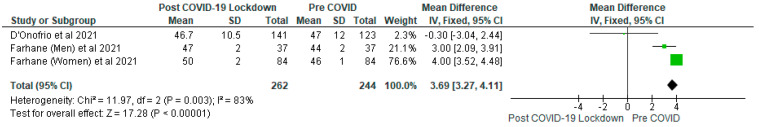
The effect of COVID-19 lockdown on HDL Cholesterol (mg/dL).

The meta-analysis of the HDL cholesterol (mg/dL) involved only two studies with 506 participants (post-COVID-19 lockdown gp, *n* = 262; pre-COVID gp, *n* = 244). There was a significantly (*p* < 0.05) higher level of HDL cholesterol in the post-COVID-19 lockdown gp compared with pre-COVID gp with a mean difference of 3.69 (95% CI: 3.27, 4.11) (Figure 9).

### 4.4. Body Mass Index (BMI)

**Figure 10:**
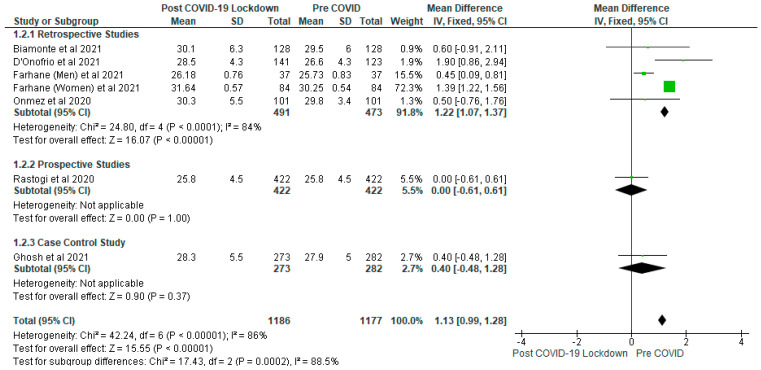
The effect of COVID-19 lockdown on Body Mass Index (kg/m^2^).

The systematic review showed that COVID-19 lockdown significantly (*p* < 0.05) increased BMI (kg/m^2^) in some studies [14,22]. The meta-analysis of the body mass index also demonstrated that post-COVID-19 lockdown gp had a significantly (*p* < 0.05) higher BMI than the pre-COVID gp with a mean difference of 1.13 (95% CI: 0.99; 1.28) (Figure 10). The result remained consistent when each study was removed one by one from the meta-analysis. Six studies were included in the analysis, involving a total of 2363 participants (post-COVID-19 lockdown gp, *n* = 1186; pre-COVID gp, *n* = 1177). Similar results were obtained in the subgroup analysis involving the retrospective studies, although differences between the post-COVID-19 lockdown gp and pre-COVID gp were not significant (*p* > 0.05) in respect of the prospective and case control studies (Figure 10).

## 5. Discussion

The results of the systematic review and meta-analysis have shown that COVID-19 lockdown significantly (*p* < 0.05) increased the levels of glycated haemoglobin (%), fasting glucose (mg/dL), HDL cholesterol (mg/dL) and body mass index (kg/m^2^) in patients with type 2 diabetes. In contrast, the lockdown significantly (*p* < 0.05) reduced postprandial blood glucose (mg/dL), total cholesterol (mmol/L), triglycerides (mmol/L) and LDL cholesterol (mmol/L).

The findings of this review in respect of the blood glucose parameters would appear to confirm the result of a previous systematic review [31] which reported that the COVID-19 lockdown resulted in short-term worsening of glycaemic control in patients with type 2 diabetes. However, the result is different from the outcome of a previous meta-analysis of observational studies [17] which showed that COVID-19 lockdown had no detrimental effect on glycated haemoglobin in either patients with type 1 diabetes or type 2 diabetes, and that it led to a reduction in mean glucose and glucose variability in patients with type 1 diabetes. Variations in the designs of the studies and differences in the countries where the studies were conducted may explain the differences in the findings of our review compared with the previous review by Silverii et al. [17]. Furthermore, the inclusion of studies involving people with type 1 and type 2 diabetes was a primary difference between our review and the earlier reviews [17,31].

The results obtained in this review in relation to poor glycaemic control and increased body mass index during the COVID-19 lockdown in patients with type 2 diabetes could be due to changes in lifestyle, including reduced physical activities and poor eating behaviours, which resulted in weight gain and subsequent loss of glucose homeostasis. Furthermore, restrictions on medical monitoring and non-compliance with diabetic management guidelines may have impacted on glycemic control during the lockdown [9]. Increased stress levels during the COVID-19 lockdown could also have led to hyperglycaemia through involvement in unhealthy behaviours, such as binge eating, reduced physical activities and the production of stress hormones, including cortisol, glucagon and the development of low-grade inflammation [21]. For example, Ruissen et al. [11] found that in people with well-controlled type 2 diabetes, the COVID-19 and lockdown measures resulted in increased stress, weight gain and decreased physical activities, although these did not lead to deterioration in glycaemic control. However, Ruissen et al. [11] also observed that changes in daily activities and behaviours can influence diabetes self-management and glycemic control.

Stress and lifestyle changes have been associated with worse glycaemic control and gain in body weight in patients with type 2 diabetes [25,32]. There is evidence of the increasing role of weight gain and the development of insulin resistance and dysregulation of glucose metabolism [22]. In terms of the relationship between weight gain, obesity and insulin resistance in patients with type 2 diabetes, the mechanism appears to be related to excess lipid accumulation in the liver [33]. It has been suggested that excessive lipid accumulation may lead to insulin signalling via autonomous mechanisms in the cells or through the production of inflammatory cytokines by macrophages which impair insulin action [34].

The regulation of normal blood glucose at rest and during exercise is mostly controlled by the sympathetic nervous system and endocrine system [35]. Skeletal muscles when at rest prefer to utilise free fatty acids as sources of energy, especially between meals [34]. However, exercise leads to the use of a combination of free fatty acids, circulating glucose and glycogen that is stored, and the balance between these three sources is dependent on exercise intensity and duration [34]. With the depletion of the glycogen store, the muscles increase their uptake and utilisation of circulating blood glucose and free fatty acids released from the adipose tissue [35]. Furthermore, with increasing exercise intensity, there is a greater reliance on carbohydrate as a source of energy [35].

In particular, skeletal muscle contraction stimulates glucose transport and metabolism via insulin independent pathway, and exercise can also promote the ability of insulin to activate glucose transport in muscles [34]. Therefore, regular exercise can stimulate the synthesis of components required for glucose uptake and metabolism in the muscles, such as Glut4 glucose transporter and hexokinase [34]. These activities support the clearance of glucose from the circulation and the metabolism of glucose in exercised muscles, including the utilisation of glucose during exercise and the re-synthesis of glycogen after exercise [34]. Therefore, glycated haemoglobin and fasting blood glucose, which are indicators of glucose control, are lowered following regular exercise [34].

However, glycated haemoglobin, fasting blood glucose and postprandial blood glucose are different biochemical measures of blood glucose levels in patients with diabetes and may offer explanations for the outcome of the results obtained in this review. While fasting blood glucose is the amount of the blood glucose level measured before breakfast and after at least 8 h of fasting [36], the postprandial glucose is usually a measure of blood glucose level after a meal. The most well-known postprandial glucose measure is the 2 h oral glucose tolerance test for the diagnosis of diabetes which involves the administration of 75 g oral glucose load [37,38]. In contrast, the glycated haemoglobin is a measure of the average glycaemia over the preceding period of about 8 weeks and it is often used to assess long term glycaemic control [36,39]. Glycated haemoglobin reflects an integrated summary of circadian blood glucose concentrations [39]. Differences could also be due to the number of studies included in the postprandial glucose analysis; only 3 studies compared to 11 studies in glycated haemoglobin analysis and 6 in the fasting plasma glucose analysis.

Several studies have suggested that inadequately controlled blood glucose is a risk factor for poor clinical outcomes in patients with type 2 diabetes and COVID-19 [22,40,41]. Therefore, it is essential that effective self-management strategies are promoted in patients with type 2 diabetes. Biamonte et al. [22] observed that the COVID-19 lockdown measures exacerbated all the risk factors for weight gain, including unfavourable eating habits and changes in lifestyle in patients with type 2 diabetes.

The variation in dietary habits of participants in the different studies included in this review may have accounted for the results obtained with respect to the lipid parameters [23]. For example, it has been shown that high carbohydrate diets may increase plasma triglycerides and decrease HDL cholesterol [23,42] in patients with type 2 diabetes. This view is further strengthened by the results of the sensitivity analysis which demonstrated that removing the Farhane et al. [9] study, there was no significant difference (*p* > 0.05) between the post-COVID-19 gp and pre-COVID gp concerning total cholesterol, triglyceride, LDL and HDL cholesterol. Furthermore, the individual studies included in the meta-analysis did not show any significant differences between the post-COVID-19 gp and pre-COVID gp with respect to lipid parameters (Figure 6, Figure 7 and Figure 8), except the Farhane et al. [9] study.

## 6. Limitations

A significant number of the studies included in this review were retrospective studies that were based on available records and may be prone to bias in terms of the participants included in these studies. This may have implications in relation to the consistency of the findings of the review.

## 7. Conclusions

The findings of the systematic review and meta-analysis have demonstrated that COVID-19 lockdown resulted in a significant increase (*p* < 0.05) in the levels of glycated haemoglobin, fasting glucose and body mass index in patients with type 2 diabetes. In contrast, the effect of the lockdown on lipid parameters, including total cholesterol, triglycerides, LDL and HDL cholesterol was not consistent. More prospective studies are needed to further elucidate the impact of the COVID-19 lockdown on glycaemic control and lipid parameters in patients with type 2 diabetes.

## Figures and Tables

**Table 1:** Search Terms and Search Strategy.

Patient/Population	Intervention	Outcome (Primary)	Combining Search Terms
Patients with type 2 diabetes	COVID-19 Lockdown	Glycaemic and other metabolic parameters	
Patients with diabetes OR Type 2 diabetes OR Diabetes OR Diabetes complications OR diabetes mellitus, type 2 OR diabetes mellitus	Corona virus OR COVID-19 OR COVID-19 testing OR SARS-CoV-2	Fasting blood glucose OR Glycated haemoglobin OR HbA1c OR Diabetic complications OR diabetic ketoacidosis	Column 1 AND Column 2 AND Column 3

**Table 2:** The description and characteristics of included studies.

Citation/Country of Study	Type of Study	Sample Details	Mean Age (Years)	Aim	Study Design/Method	Results
Biamonte et al. [22] Italy	Retrospective, observational study	128 participants with type 2 DM aged 18 yrs and above	40–91	To evaluate the impact of COVID-19 lockdown in Italy from 9 March to May 18 2020 on anthropometric parameters and glycaemic control in patients type 2 DM	A retrospective, observational study based on medical records. Evaluation was based on baseline between 15 December 2019 to 1 March 2020 compared to post lockdown between 15 and 30 June 2020	During lockdown, there were significant increases in the following: body weight from 79.7 ± 18.7 kg to 81.4 ± 19.4 kg, *p* < 0.001; BMI from 29.5 ± 6 kg/m^2^ to 30.1 ± 6.3 kg/m^2^, *p* < 0.001; Waist circumference from 103.8 ± 13 cm to 105 ± 13.6 cm *p* < 0.001; Fasting plasma glucose from 138.1 ± 29.4 mg/dl to 146.6 ± 36.4 mg dl and HbA1c from 7 ± 0.8% to 7.3 ± 0.9%, *p* < 0.001
Biancalana et al. [23] Italy	A single centre, prospective, observational study	114 participants aged <85 years	69.4 ± 10.3	To explore the short-term impact of lockdown on metabolic control in patients with well-controlled type 2 DM.	A prospective observational study which assessed patients who were previously scheduled for follow-up visit during lockdown (9 March–4 May 2020).	After eight weeks of lockdown, an increase of HbA1c > 0.3% was observed in 26% of participants and triglycerides were persistently elevated.
D’Onofrio et al. [14] Italy	Observational multicentre retrospective cohort study	264 participants with T2DM	61.0–76.0	To assess the effect of COVID-19 lockdown on glycaemic control in patients type 2 DM	An observational retrospective study consisting of 141 patients (lockdown group) and 123 patients (control group; pre-covid)	No difference in HbA1c was found in both groups (lockdown group: −0.01% [−0.5%, −0.3%] vs. control group −0.1% [−0.4%, −0.2%]; *p* = 0.482. Glucose (mg/dL) (*p* = 0.538). BMI (kg/m^2^) (*p* = 0.316)
Farhane et al. [9] Morocco	Retrospective observational study	121 patients with T2DM aged 36–85 years	57.31 ± 0.91	To analyse the impact of lockdown on monitoring and care of T2DM patients in Morocco	A retrospective observational study. Biochemical, socio-demographic and anthropometric data were collected from each patient pre-lockdown (1 November 2019–19 March 2020) and post-lockdown (6 July–29 December 2020).	Lockdown impacted negatively on health status of T2DM patients, especially women. In women, HbA1c increased from 8.66 ± 0.21% to 9.51 ± 0.25% (*p* = 0.001) HDL-C (g/L) increased too (*p* = 0.0132), weight increased from 78.13 ± 1.36 kg to 81.80 ± 1.45 kg with *p* < 0.000, Systolic blood pressure (mmHg) reduced after lockdown (*p* = 0.0302).
Ghosh et al. [21] India	Case control	555 participants aged 18 and above with new onset of diabetes	46.2 ± 12.3	To investigate if new onset diabetes during COVID-19 is phenotypically or biochemically different from new onset diabetes before COVID-19	Patient diagnosed from 1 April 20–30 October 20 (Covid group) from two hospitals were in one group and patients diagnosed from 1 September 19–29 February 20 (pre-covid) from same hospital were in comparator group.Data were collected and compared.	There was no significant difference in symptomatology, phenotype and C-peptide levels between the pre-covid and Covid groups but the Covid group had more hyperglycaemia probably due to delayed diagnosis.
Ludwig et al. [24] France	Observational cross-sectional single-centre study	870 adults living with type 1 or type 2 diabetes	65.0 (57.0, 72.0)	To evaluate the impact of the COVID-19 lockdown on metabolic control and access to healthcare in patients with diabetes.	Data were collected from existing medical records and self-administered questionnaire from patients pre (18 September 2019 to 24 March 2020) and post (11 May 2020 to 20 June 2020) lockdown. These data were compared.	Despite the lockdown and disruption in healthcare, there was improvement in metabolic control in a large sample of patients.HbA1c pre-lock was 7.7% (7.1, 8.4) and post-lockdown was 7.4% (6.8, 8.2) (*p* < 0.0001).
Munekawa et al. [25] Japan	cross-sectional and retrospective cohort study	203 patients with T2DM	67.4 (11.3)	To investigate the acute effects of the COVID-19 pandemic on the lifestyle changes in patients with T2DM	Data regarding the body weight and HbA1c levels were collected from medical records and questionnaire was administered to the patients with type 2 diabetes mellitus who visited the clinic from 16 April to 1 May 2020	Results showed increased stress levels and changes in life-style factors during the COVID-19 pandemic. These lifestyle changes were associated with increased body weight and HbA1c levels. [57.9 (±10.6) to 59.7 (±12.0) mmol/mol] (*p* = 0.001).
Onmez et al. [26] Turkey	Single-centre retrospective, observational study.	101 type 2 DM patients aged 18–80 years	55 ± 13 years	To investigate how type 2 DM patients were affected by the lockdown	A retrospective, observational study was conducted between 16 March and 1 June 2020 with patients unable to attend follow-ups due to lockdown, but who attended follow-ups in July and August post lockdown	Glycaemic parameters, HbA1c increased from 7.67 ± 1.76% to 8.11 ± 2.48%, fasting glucose from 157.9 (83–645) mg/dL to 163.2 (84–550) mg/dl, postprandial glucose from 228.8 ± 72.9 mg/dl to 260.3 ±90.8 mg/dL.The changes between pre and post lockdown were not statistically significant (*p* = 0.253, *p* = 0.678 and *p* = 0.079, respectively).
Park et al. [27] South Korea	Retrospective cohort study	20,087 adult patients with T2DM. Aged 19 and over	(62.8 years) [19 to 95 years]	To determine the effects of social distancing due to COVID-19 on the changes in HbA1c level in people with T2DM.	Data were collected from the COVID-19 cohort (2019 to 2020), non-COVID-19 cohort 1 (2018 to 2019) and cohort 2 (2017 to 2018), and categorized into Periods 1 and 2. The HbA1c values for each patient were collected from their electronic medical records. The changes in HbA1c between Periods 1 and 2 in the COVID-19 cohort were compared with those in the non-COVID-19 cohorts as control groups.	Social distancing due to COVID-19 negatively impacted glycaemic control in people with T2DM
Rastogi et al. [28] India	prospective, observational, cohort study	422 patients living with T2DM	58.0 (52.0 to 64.0)	To examine the effect of lockdown on physical activity and glycaemic control in T2DM	Data relating to changes in glycaemic control (HbA1c) with the modification of weight, BMI, and physical activity during the lockdown period were collected. The pre- and post-lockdown glycaemic variables were compared using Wilcoxon signed-rank *t* test	Result showed that there is an overall improvement of glycaemic control during COVID-19 lockdown independent of increase in physical activity in people with long duration of T2DM. HbA1c pre lockdown was 7.8% (6.9 to 9.4%) compared with 7.4% (6.6 to 8.7) after 3 months of lockdown (*p* = 0.005)
Sankar et al. [29] India	Hospital-based cross-sectional survey	110 Adult participants with T2DM	58.67 ± 10.8 years.	To identify the effects of lockdown on glycaemic status, lifestyle changes and psychosocial health.	The pre- and post-lockdown data of 110 adults with T2D who were under regular follow up was collected by direct interview during their visit to the diabetes clinic. The variables analysed included demographic data, HbA1c, body weight, lifestyle changes, psychosocial factors and use of technology	Lockdown did not cause a major change in the overall glycaemic control.There was no statistically significant difference in the mean HbA1c before (8.2 ± 1.3%) and after (8.12 ± 1.6%) lockdown. The mean body weight after the lockdown was numerically higher (71.8 ± 13.6 kg) compared to that before lockdown (71.5 ± 14.8 kg), but could not achieve statistical significance.

Abbreviations: DM (Diabetes mellitus); T2DM (Type 2 diabetes mellitus).

## Data Availability

We conducted secondary data analysis of publicly available data.
